# The Multi-Template Molecularly Imprinted Polymer Based on SBA-15 for Selective Separation and Determination of *Panax notoginseng* Saponins Simultaneously in Biological Samples

**DOI:** 10.3390/polym9120653

**Published:** 2017-11-28

**Authors:** Chenghong Sun, Jinhua Wang, Jiaojiao Huang, Dandan Yao, Chong-Zhi Wang, Lei Zhang, Shuying Hou, Lina Chen, Chun-Su Yuan

**Affiliations:** 1 School of Pharmacy, Nanjing Medical University, Nanjing 211166, China; sbh1173250588@163.com (C.S.); nydhjj_celia@163.com (J.H.); 18851726822@163.com (D.Y.); 18851720730@163.com (L.Z.); 2 Department of Pharmacy Intravenous Admixture Service, The First Affiliated Hospital of Harbin Medical University, Harbin 150001, China; wangjinhua.txd@126.com; 3 Tang Center for Herbal Medicine Research and Department of Anesthesia & Critical Care, University of Chicago, Chicago, IL 60637, USA; cwang@dacc.uchicago.edu (C.-Z.W.); cyuan@dacc.uchicago.edu (C.-S.Y.)

**Keywords:** multi-template molecularly imprinted polymers, SBA-15, *Panax notoginseng* saponins, separation and determination, solid-phase extraction

## Abstract

The feasible, reliable and selective multi-template molecularly imprinted polymers (MT-MIPs) based on SBA-15 (SBA-15@MT-MIPs) for the selective separation and determination of the trace level of ginsenoside Rb_1_ (Rb_1_), ginsenoside Rg_1_ (Rg_1_) and notoginsenoside R_1_ (R_1_) simultaneously from biological samples were developed. The polymers were constructed by SBA-15 as support, Rb_1_, Rg_1_, R_1_ as multi-template, acrylamide (AM) as functional monomer and ethylene glycol dimethacrylate (EGDMA) as cross-linker. The new synthetic SBA-15@MT-MIPs were satisfactorily applied to solid-phase extraction (SPE) coupled with high performance liquid chromatography (HPLC) for the separation and determination of trace Rb_1_, Rg_1_ and R_1_ in plasma samples. Under the optimized conditions, the limits of detection (LODs) and quantitation (LOQs) of the proposed method for Rb_1_, Rg_1_ and R_1_ were in the range of 0.63–0.75 ng·mL^−1^ and 2.1–2.5 ng·mL^−1^, respectively. The recoveries of R_1_, Rb_1_ and Rg_1_ were obtained between 93.4% and 104.3% with relative standard deviations (RSDs) in the range of 3.3–4.2%. All results show that the obtained SBA-15@MT-MIPs could be a promising prospect for the practical application in the selective separation and enrichment of trace *Panax notoginseng* saponins (PNS) in the biological samples.

## 1. Introduction

*Panax notoginseng* (Burk.) F. H. Chen (*P. notoginseng*) is a species of the genus *Panax*, family Araliaceae, which has been officially recorded in USP Herbal Medicines Compendium and China Pharmacopeia [1,2]. The radix and rhizome of *P. notoginseng*—known as Sanqi or Tianqi in East Asian countries—is one of the primary herbs in natural products. Sanqi has wide-ranging pharmacological effect such as anti-inflammation [3], cardioprotective effect [4], anticarcinogenic effect [5], anti-atherosclerotic [6] as well as antioxidant [7], etc. The major bioactive constituents responsible for these pharmacological effects are extensively recognized as the *Panax notoginseng* saponins (PNS), among which ginsenoside Rb_1_ (Rb_1_), ginsenoside Rg_1_ (Rg_1_) and notoginsenoside R_1_ (R_1_) are three main components and thus generally considered as quality control markers in the manufacturing of Sanqi related preparations [2,8]. The content and their ratios of PNS in Sanqi are also strongly correlated to its quality and efficacy [9]. In the current scenario, a few studies on the toxic effects of PNS due to unstable components, impurities in prescriptions and PNS over dose have appeared during clinical treatments [10,11,12]. Until now, various methods have been already proposed to assay PNS, including high performance liquid chromatography-electrospray ionization mass spectrometry (HPLC-ESI-MS) [13], gas chromatography-mass spectrometry (GC-MS) [14], micellar electrokinetic chromatography (MEKC) [15] and enzyme-linked immunosorbent assay (ELISA) [16]. However, because of multi-components, extremely low concentrations of PNS and the complex nature of the matrices in complex samples [17], it is almost impossible to directly identify PNS without time-consuming pre-treatment. Currently, different attempts have been proposed and investigated, which included liquid-liquid extraction (LLE) [18], ultrafiltration (UF) membrane [19], microdialysis [20], etc. Although these pre-treatment techniques have been applied to extract a wide variety of compounds, these protocols involve some disadvantages, such as severe interference, low enrichment efficiency and limited sensitivity, etc. Therefore, it is highly desirable to establish a feasible, selective and sensitive analytical method for extraction, enrichment and quantification of major active components in body fluids to ensure the safety, efficacy and stability of clinical application of PNS. 

Molecular imprinting technology (MIT), often described as a method of making a molecular lock to match a molecular key, is a technique for the creation of molecular imprinting polymers (MIPs) with tailor-made recognition sites complementary to template molecules in shape, size and functional groups [21]. The recognition sites are generated by a process that involves co-polymerization of functional monomers and cross-linkers around template molecules [22]. Interestingly enough, templates are removed from the polymers rendering complementary binding sites capable of subsequent template molecule recognition [23,24]. Due to their unique features of high specificity, mechanical strength and resistance against organic solvent, high pressure and temperature, MIPs have gained enormous application in electrochemical sensor [25], solid-phase extraction [26] and biomimetic catalysis [27], etc. Regretfully, traditional bulk MIPs exhibit some disadvantages, including incomplete template removal, poor site accessibility to target species and irregular shape, which is bound to affect the rebinding and selective recognition of target molecules. The surface imprinting technology can overcome the above problems effectively. Recently, mesoporous silica has been proven to be an excellent matrix material that possesses high hydrothermal stability, tunable pore sizes, ordered mesoporous structure and large specific surface areas [28], which can significantly enhance the adsorption capability of porous adsorbents. SBA-15 with uniform hexagonal channels is one of the mesoporous silica molecular sieves. Compared with other mesoporous materials, such as MCM-41, SBA-15 possesses ultra large pore size, thick walls and better hydrothermal stability [29,30]. Based on SBA-15, the present study was focused on synthesizing novel MIPs endowed with higher specific surface area, more accessible binding site and faster mass transfer rate. 

In our initial studies, we synthesized highly selective single-template MIPs based on mesoporous materials for selective separation and quantitative analysis of natural products in biological specimens [31,32]. As we all know, the single-template MIPs have recognition sites only for one target molecule, which leads to poor selectivity and affinity for a family of analytes in most cases [33,34], except that experimental conditions were optimized, such as the optimization of extraction process [35]. Furthermore, the single-template MIPs are not efficient in the case of recognition of various targeted analyses simultaneously from complex samples [36]. In addition, physically mixing individually imprinted polymers are also not feasible as synthesizing several polymers require considerable time and effort [34]. Actually, by using several targets/species as templates, different classes of species can be extracted, separated, assayed and detected simultaneously, which can increase their utility and expand their potential application [34]. To circumvent the fundamental defects of single-template MIPs, multi-template MIPs (MT-MIPs) prepared with two or multiple templates, which can simultaneously absorb a group of structural analogues, are highly desirable for sustainable development. However, there is no publication to date that reports the simultaneous separation and enrichment of Rb_1_, Rg_1_ and R_1_ by MT-MIPs based on SBA-15 (SBA-15@MT-MIPs) in biological samples.

In this study, we prepared highly sensitive and selective polymers as a novel and inexpensive sorbent for the isolation and enrichment of Rb_1_, Rg_1_ and R_1_ in biological samples. The SBA-15@MT-MIPs were synthesized using R_1_, Rb_1_, Rg_1_ as multi-template, acrylamide (AM) as functional monomer, ethylene glycol dimethacrylate (EGDMA) as cross-linker and ethanol as porogen. The resulting products were characterized by TEM, FT-IR, TGA, N_2_ adsorption-desorption analysis. Adsorption properties of obtained SBA-15@MT-MIPs were investigated, which included adsorption isotherm, adsorption kinetics and selective recognition. In addition, a molecularly imprinted solid-phase extraction (MISPE) procedure coupled with HPLC was successfully developed to isolate and detect trace amounts of Rb_1_, Rg_1_ and R_1_ in rat plasma samples, which indicated that the novel SBA-15@MT-MIPs can provide a proper example of an analytical method for quality control, support pharmacological and clinical studies of PNS.

## 2. Materials and Methods

### 2.1. Chemicals and Reagents

Ginsenoside Rg_1_ (Rg_1_), ginsenoside Rb_1_ (Rb_1_), notoginsenoside R_1_ (R_1_), ursolic acid (UA), oleanolic acid (OA) were supplied by the National Institute for the Control of Pharmaceutical and Biological Products (Beijing, China). Poly (ethylene glycol)-block-poly (propylene glycol)-block-poly (ethyiene glycol) (P123), tetraethoxy silane (TEOS), tetrahydrofuran, ethylene glycol dimethacrylate (EGDMA) and 4-vinylpyridine (4-VP) were purchased from Sigma-Aldrich (Steinheim, Germany). Acrylamide (AM), methacrylic acid (MAA) and 2,2-azobisisobutyronitrile (AIBN) from Sinopharm (Shanghai, China). 3-Methacryloxypropyltrimethoxysilane (MPS) was obtained from Aladdin (Shanghai, China). Acetonitrile of HPLC grade was prepared from Merck (Darmstadt, Germany). Ultrapure water (18.2 MΩ cm) was produced using a Millipore water purification system (Merck Millipore, Darmstadt, Germany) and used for the entire experiment. All other chemicals used in this study were of the analytical reagent grade. 

The powder of *Panax notoginseng* was donated by the Department of Pharmacognosy, Nanjing Medical University (Nanjing, China).

### 2.2. Apparatus

The FT-IR analysis was conducted in the form of KBr pellets and using a Fourier Transform Infrared (FT-IR) spectrometer (Bruker, Switzerland) in a range of 500–4000 cm^−1^. Ultraviolet-Visible (UV-Vis) absorbance detection was achieved by a Shimadzu UV-2100 spectrophotometer (Shimadzu, Kyoto, Japan). Heating magnetic stirrer (C-MAG HS 7, IKA, Staufen, Germany) was applied during the preparation. The morphologies and structures of polymers were observed by a scanning electron microscope (SEM, FEI Quanta 200, FEI, Hillsboro, OR, USA) and a transmission electron microscope (TEM, JEM-2100, JEOL, Tokyo, Japan). A shake culture box (ZHLY-180, Shanghai, China) was applied during the binding experiment. N_2_ adsorption-desorption analysis was carried out at 77 K using a Micromeritics ASAP 2010 system (Micromeritics, Norcross, GA, USA). Thermogravimetric analysis was conducted on a thermogravimetric analyzer (TGA, Pyris 1 DSC, Perkin-Elmer, Waltham, MA, USA) with a heating rate of 20 °C·min^−1^ from 25 °C to 700 °C under a stream of N_2_ gas (200 mL·min^−1^). HPLC analysis was performed with a Shimadzu (Kyoto, Japan) system comprising LC-20AT pump, SPD-M 20A detector, CTO-20A column oven and HW-2000 chromatographic work station. The structural identification of target compounds was performed using an Agilent 1200 liquid chromatograph (Agilent, Santa Clara, CA, USA) coupled with an Agilent 6410B Triple Quad mass spectrometer.

### 2.3. Synthesis of SBA-15 Support and Modified SBA-15

#### 2.3.1. Synthesis of SBA-15 Substrate

The SBA-15 was synthesized according to the previous literature [33]. In brief, in a 250 mL round-bottomed flask, 2.0 g P123 was mixed with 2.0 mmol·L^−1^ hydrochloric acid (60 g), 0.033 mol acetic acid and 0.83 mol water under vigorous stirring at 40 °C. Subsequently, 4.25 g TEOS was added into the abovementioned solution drop by drop under vigorous stirring for 24 h at 40 °C and the mixture was transferred into Teflon-lined autoclaves and heated at 100 °C for 48 h. Finally, the resulting precipitates were filtered and washed with distilled water and then dried at ambient temperature. The organic templates were removed by calcining in air for 6 h at a heating rate of 5 °C·min^−1^ up to 550 °C.

#### 2.3.2. Preparation of Vinyl Modified SBA-15

The surface of the as-prepared SBA-15 was modified by MPS according to our previous report [31]. Briefly, 500.0 mg SBA-15, 10 mL MPS and 50 mL anhydrous toluene were placed into a 100 mL three-necked flask and stirred. Then, the mixture solution was degassed with N_2_ for about 10 min followed by heating the mixture to 55 °C for 24 h. Finally, the modified SBA-15 (SBA-15-MPS) containing vinyl terminal group was centrifuged, washed with toluene and methanol successively and dried under vacuum at 45 °C for 24 h.

### 2.4. Imprinting at the Surface of Modified SBA-15

**Figure 1:**
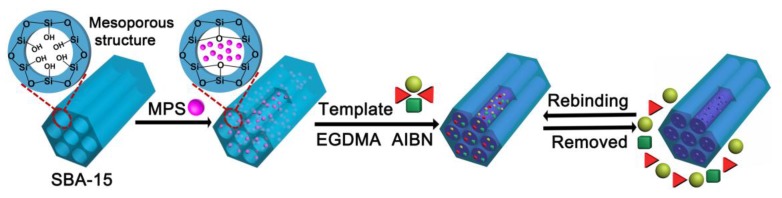
Schematic representation for the preparation of the SBA-15@MT-MIPs.

The SBA-15@MT-MIPs were prepared by surface molecular imprinting technique, as shown in Figure 1. Here, a facile free-radical polymerization method was employed. Prior to polymerization, 0.2 mmol of the mixture of Rb_1_, Rg_1_ and R_1_ (the ratio of Rb_1_ to Rg_1_ to R_1_ was 44:43:13 presented in *P. notoginseng*) [37] and functional monomer were dissolved in 30 mL of solvent, which was incubated for 4 h at ambient temperature for pre-polymerization to prepare the prearranged solution. Then 0.1 g SBA-15-MPS was dispersed in 20 mL of solvent by ultrasonic vibration. The prearranged solution, cross-linker (EGDMA) and initiator (AIBN) (the weight was about 5% of the total mass of templates, functional monomers and cross-linkers) were added to the above solution and mixed. The mixture was de-aerated by N_2_ for 20 min and carried out in a shake culture box at 60 °C for 24 h. After the reaction, the SBA-15@MT-MIPs were centrifuged and washed successively with methanol to remove all absorbed oligomers and unreacted monomers. Finally, the SBA-15@MT-MIPs were washed repeatedly with methanol-acetic acid (9:1, *v*/*v*) to remove the templates until no templates in the supernatant were detected using a UV spectrophotometer. The resulting imprinted polymers were repeatedly washed with methanol until the supernatant was neutral and then dried in a vacuum at 40 °C for 24 h. The multi-template non-imprinted polymers based on SBA-15 (SBA-15@MT-NIPs) were also fabricated and purified under identical condition except that the templates were omitted.

### 2.5. Evaluation of the Binding Ability of SBA-15@MT-MIPs 

To evaluate the adsorption performance of SBA-15@MT-MIPs, the isotherm, kinetics and selective adsorption experiments were carried out. All experiments were repeated three times in parallel. In all experiments, 20 mg polymers were dispersed in a 5 mL ethanol solution. The mixture was incubated on a shaker at 200 rpm for a period of time and then filtered using a 0.22 μm microporous membrane. The maximum absorbance of Rb_1_, Rg_1_ and R_1_ at about 204 nm before and after adsorption was measured by UV-Vis spectrophotometer and the corresponding concentrations were calculated through the calibration curve. The adsorption capacity (*Q*, μmol·g^−1^) of the template bound to the polymer was calculated by the following Equation (1):

(1) $$Q_{e}=\frac{(C_{0}-C_{e})V}{m}$$

where *Q_e_* is the equilibrium adsorption amount of Rb_1_, Rg_1_ and R_1_, *C*_0_ and *C*_e_ represent the initial concentration and the equilibrium concentration of Rb_1_, Rg_1_ and R_1_, respectively (mmol·L^−1^), *V* is the volume of solution tested and *m* is the mass of the polymer.

In order to investigate the recognition performance of adsorbents for target molecules, an equilibrium adsorption experiment was studied at various concentrations of the mixture solution of Rb_1_, Rg_1_ and R_1_ ranging from 0.5 mmol·L^−1^ to 4.0 mmol·L^−1^. The mixture was kept shaking for 80 min at ambient temperature.

To investigate the adsorption rate of the obtained SBA-15@MT-MIPs, an adsorption kinetics experiment was conducted. The adsorption time was changed at regular intervals from 0 min to 80 min, while the mixture solution of Rb_1_, Rg_1_ and R_1_ was kept at a concentration of 3.0 mmol·L^−1^, respectively.

To measure the specificity of SBA-15@MT-MIPs, UA and OA were adopted as structural analogues to compare with Rb_1_, Rg_1_ and R_1_. A selective adsorption experiment was investigated using solutions of R_1_, Rg_1_, Rb_1_, UA and OA at a concentration of 3.0 mmol·L^−1^ and incubation time of 60 min. The imprinting factor (*IF*) was used to evaluate the specific recognition property of the polymers towards the targets and structural analogues, which was calculated by Equation (2):

(2) $$IF=\frac{Q_{MIP}}{Q_{NIP}}$$

here *Q_MIP_* and *Q_NIP_* are the adsorption capacities of the template and/or structural analog on SBA-15@MT-MIPs and SBA-15@MT-NIPs, respectively.

To investigate the stability and reusability of SBA-15@MT-MIPs, six adsorption-desorption cycles were conducted by using the same SBA-15@MT-MIPs. The adsorption procedure was the same as the binding experiment. The concentration of the mixture solution of Rb_1_, Rg_1_ and R_1_ and incubation time was 3.0 mmol·L^−1^ and 60 min, respectively. Elution of Rb_1_, Rg_1_ and R_1_ from SBA-15@MT-MIPs was performed using methanol-acetic acid (9:1, *v*/*v*) and methanol in turn.

### 2.6. Separation and Determination of Rb_1_, Rg_1_ and R_1_ in Real Samples

#### 2.6.1. Preparation of Plasma Samples

50.0 g of *P. notoginseng* powder was weighted accurately and placed into a 500 mL round-bottom flask and 400 mL of 70% ethanol was added. The mixture was refluxed for 4 h at 80 °C. The extraction was repeated two additional times and the combined extracts were concentrated to about 10 mL under vacuum and then diluted to 50 mL with physiological saline. Ten male SD rats (250–280 g) were fed with standard laboratory food and water ad libitum except for fasting 12 h prior to experiment. The extract solution was orally administered to rats at the doses equivalent to 10 g·kg^−1^ of the powder of *P. notoginseng* and equal volume of normal saline for the blank group. At 50 min after drug dosing, orbital blood samples were collected into heparinized microfuge tubes and immediately centrifuged at 3000 rpm for 15 min to obtain plasma, which was precisely collected and stored at −20 °C until analysis.

In order to reduce complexity of plasma matrix, the extraction was carried out by adding quadruple volume of methanol to the plasma and the mixture was vortexed for 30 s and then centrifuging at 12,000 rpm for 10 min to precipitate the denatured proteins. The supernatant was evaporated to dryness under a gentle N_2_ stream at room temperature and then re-dissolved with 5 mL toluene-ethanol (7:3, *v*/*v*) and collected as analytical samples.

#### 2.6.2. Enrichment of Rb_1_, Rg_1_ and R_1_ from Plasma Samples by MISPE 

Firstly, 500 mg of SBA-15@MT-MIPs and SBA-15@MT-NIPs were packed manually in an empty solid-phase extraction (SPE) column. Each cartridge was rinsed with methanol-acetic acid (9:1, *v*/*v*) and no template was detected in the obtained solution by HPLC. After being conditioned with 5 mL toluene-ethanol (7:3, *v*/*v*), 5 mL of analytical samples were loaded into the cartridge and sealed. Subsequently, it was washed with 5 mL of washing solution to remove the interfering substances and then was dried thoroughly by a vacuum pump. Finally, it was eluted with 5 mL of methanol-acetic acid (9:1, *v*/*v*) to get the desired Rb_1_, Rg_1_ and R_1_. Both the washing and elution fractions were evaporated to dryness under a gentle N_2_ stream at room temperature and the obtained residues were re-dissolved in 1 mL methanol for HPLC analysis.

#### 2.6.3. Chromatographic Analysis

The amount of Rb_1_, Rg_1_ and R_1_ was analyzed by an HPLC system. A Shimadzu shim-pack C_18_ column (250 mm × 4.6 mm, 5 μm) was used as the stationary phase. The mobile phase consisted of ultra-water (A) and acetonitrile (B) and was run in an isocratic mode at a flow rate of 1.0 mL·min^−1^. The following gradient was performed: 0–23 min, 23% B; 23–40 min, 35% B. The column temperature was maintained at 35 °C. Aliquots of 20 μL were injected. Monitoring and quantitation of Rb_1_, Rg_1_ and R_1_ were performed at 204 nm. 

Agilent 1200 liquid chromatograph combined with an Agilent 6410B Triple Quad mass spectrometer was used to identify Rb_1_, Rg_1_ and R_1_ in biological samples. An Agilent zorbax eclipse plus C_18_ column (150 mm × 2.1 mm, 5 μm) was used as the stationary phase. The mobile phase for LC-MS analysis consisted of acetonitrile-water (5:5, *v*/*v*) at a flow rate of 0.5 mL·min^−1^.

## 3. Results

### 3.1. The Optimization of Preparation Conditions of SBA-15@MT-MIPs

The successful preparation of MIPs largely depends on the judicious selection of the functional precursor, such as functional monomer, solvent/porogen and their relative molar ratio, etc. So, the preparation conditions of SBA-15@MT-MIPs should be optimized.

The selectivity of SBA-15@MT-MIPs is closely related to the number and shape of binding sites within the polymer network structure, as well as the rigidity of the complexes formed by template molecule and functional monomer [38]. Therefore, monomer selection is the first priority for the synthesis of SBA-15@MT-MIPs with good affinity and high selectivity. In this study, three potential functional monomers (4-VP, AM and MAA) were screened to prepare the polymers. In Table 1, SBA-15@MT-MIPs-3 showed the highest *Q* (108.9 µmol·g^−1^) and *IF* values (2.11). It was concluded that the monomer based on AM created SBA-15@MT-MIPs with higher affinity binding sites for Rb_1_, Rg_1_ and R_1_ compared to other study monomers. Although the nitrogen of pyridine group in the 4-VP and the oxygen of the carbonyl in the MAA and AM were likely to become the hydrogen bond acceptors for the protons of the hydroxyl groups of the Rb_1_, Rg_1_ and R_1_, the monomer based on AM created SBA-15@MT-MIPs with higher affinity binding sites for Rb_1_, Rg_1_ and R_1_ compared to other study monomers. The reason may be that strong complexes were formed between AM and templates [39]. Therefore, AM was chosen as the functional monomer in the experiment. In addition, the ratio of functional monomer to the cross-linker has an influence on binding capacity and rigid polymer network. In this study, 1:4 was the optimum molar ratio of monomer to cross-linker according to previous paper reports [40,41].

**Figure 2:**
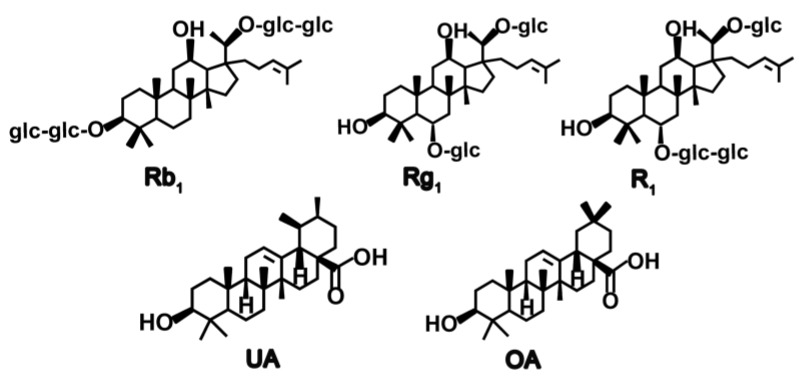
Molecular structures of Rb_1_, Rg_1_, R_1_, UA and OA.

Generally, the ratio of template to functional monomer has influence on binding affinity and imprinting effect of the polymers, so we examined the effect of different molar ratios of template to functional monomer in a range of 1:3–1:6. The molecular structures of Rb_1_, Rg_1_ and R_1_ can be seen in Figure 2. The results in Table 1 revealed that the SBA-15@MT-MIPs-3 had the highest adsorption capacity and selectivity with the molar ratios of 1:4, while other molar ratios showed lower adsorption capacity and selectivity. The reason may be that fewer templates induce fewer binding sites in polymers due to fewer template-monomer complexes but residual templates produce lower specific binding capacity and higher non-specific binding capacity [42]. So, 1:4:16 was selected as the optimum molar ratio of template to functional monomer to cross-linker and their exact molar amount was 0.2, 0.8 and 3.2 mmol, respectively.

The selectivity and affinity of synthesized SBA-15@MT-MIPs through non-covalent interaction between active sites of SBA-15@MT-MIPs and imprinted molecules are closely related to the polarity of the porogens. In addition, porogens have a substantial impact on the self-assembly of template-functional monomer and the size of mesoporous and macropores in the particles. Rb_1_, Rg_1_ and R_1_ are valuable bioactive molecules but its high polarity [43]. So, the choice of solvents is very challenging. It is well known that high polarity porogenic solvents are likely to disturb the monomer-template interactions [44] but the low polar solvents could have failed to dissolve templates and functional monomer completely. Therefore, we chosen tetrahydrofuran, ethanol and ethanol-acetonitrile (2:3, *v*/*v*) as solvent. The results were shown in Table 1. When using tetrahydrofuran and ethanol-acetonitrile (2:3, *v*/*v*) as the porogens, the *Q* and *IF* of the SBA-15@MT-MIPs were not the highest. It was indicated that the hydrogen bond interaction between templates (Rb_1_, Rg_1_ and R_1_) and AM in ethanol was stronger than the other choices mentioned [45]. Rb_1_, Rg_1_ and R_1_ were sparingly soluble in ethanol-acetonitrile (2:3, *v*/*v*), whereas slightly soluble in tetrahydrofuran. Ethanol was chosen to be the polymerization solvent, in which Rb_1_, Rg_1_ and R_1_ can completely dissolve. So, SBA-15@MT-MIPs were prepared in ethanol in this study.

From all the above, the three parameters including the selection of the functional monomers, the molar ratios of template to functional monomer and the optimization of polymerization porogens have been optimized and the optimal material was SBA-15@MT-MIPs-3, which was employed for further study.

### 3.2. Characterization of SBA-15@MT-MIPs

**Figure 3:**
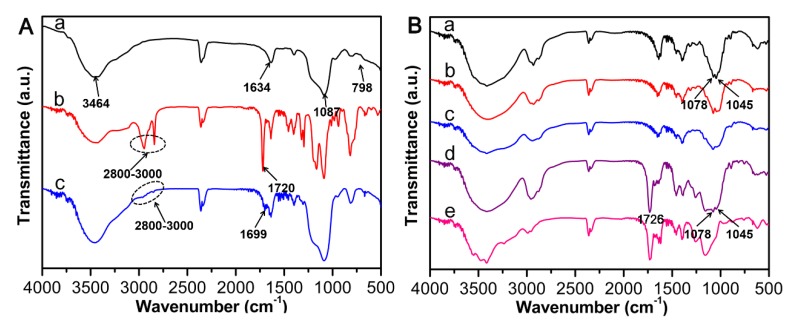
(**A**) FT-IR spectra of (a) SBA-15, (b) MPS and (c) SBA-15-MPS; (**B**) FT-IR spectra’s of (a) R_1_, (b) Rg_1_ and (c) Rb_1_, (d) SBA-15@MT-MIPs before eluting templates, (e) SBA-15@MT-NIPs.

The FT-IR spectra ascertained the successful synthesis and modification of SBA-15. Meanwhile, SBA-15@MT-MIPs had been prepared successfully. As shown in Figure 3A(a), the absorptions at 3464 cm^−1^ and 1634 cm^−1^ were ascribed to the stretching and bending vibrations of the O–H, while the peculiar peaks at 1087 cm^−1^ and 798 cm^−1^ were related to the characteristic Si–O–Si stretching vibration, indicating that SBA-15 was successfully synthesized. The peak observed at 1720 cm^−1^ indicates the presence of C=O stretching vibration and the relatively strong bands in the range of 2800–3000 cm^−1^ corresponded to the stretching vibration of C–H bonds from the methyl (or methylene) groups of MPS (Figure 3A(b)) [46]. The spectrum of SBA-15-MPS (Figure 3A(c)) showed new characteristic peak at 1699 cm^−1^, which was ascribed to the stretching vibration absorption of the C=O in ester groups. The fact suggested that mesoporous carriers were successfully modified by MPS, indicating that the vinyl-terminated SBA-15 was obtained.

The FT-IR spectra of R_1_, Rg_1_, Rb_1_, SBA-15@MT-MIPs before eluting templates and SBA-15@MT-NIPs were explored and the corresponding results were presented in Figure 3B. The as-prepared SBA-15@MT-MIPs before eluting templates (Figure 3B(d)) clearly displayed the characteristic peaks of R_1_, Rg_1_ and Rb_1_, including the peaks at 1078 cm^−1^ and 1045 cm^−1^ corresponding to [47], while the peaks were not shown in the spectrum of SBA-15@MT-NIPs (Figure 3B(e)). It can be inferred that R_1_, Rg_1_ and Rb_1_ were existed in SBA-15@MT-MIPs before elution. The peak at 1726 cm^−1^ was ascribed to the stretching vibration absorption of the C=O, which demonstrated the existence of EGDMA and AM at SBA-15@MT-MIPs and SBA-15@MT-NIPs. In conclusion, the results indicated that MIP layers were grafted onto the surface of SBA-15-MPS through the free-radical polymerization method.

**Figure 4:**
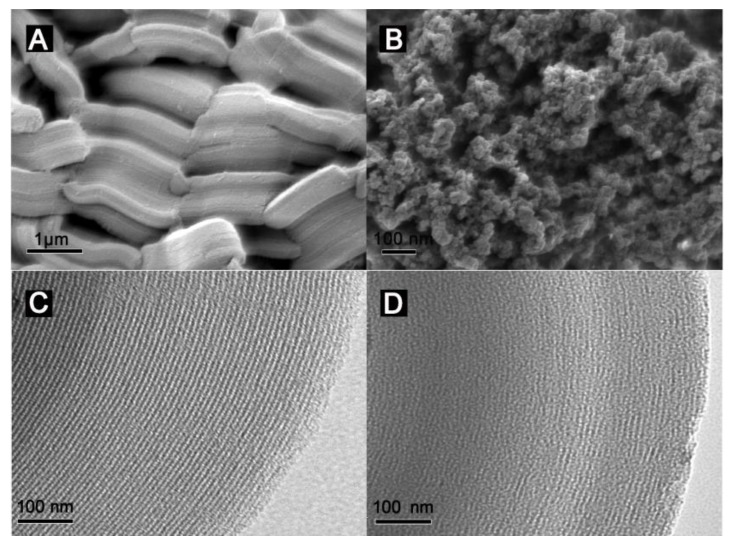
SEM images of (**A**) SBA-15 and (**B**) SBA-15@MT-MIPs; TEM images of (**C**) SBA-15 and (**D**) SBA-15@MT-MIPs.

The representative morphology and microstructure of SBA-15 and SBA-15@MT-MIPs were characterized by SEM and TEM, respectively. The size-uniformity of SBA-15 with short hexagonal rod-like structures approximately 1.4 μm were observed in Figure 4A. Compared with SBA-15, significant differences in morphology and particle shape can be seen from Figure 4B, SBA-15@MT-MIPs with a honeycomb-like form exhibited high density of macropores, which were ascribed to the generated imprinted polymer thin layers. Besides, the results confirm that agglomerated particles had nano-sized which provide suitable structure for SPE adsorbents. To investigate the morphology and microstructure thoroughly and intuitively, the TEM images of prepared materials were obtained. As can be seen in Figure 4C,D, all samples displayed the well-ordered hexagonal mesoporous structures, similarly indicating the structure of SBA-15 was maintained after modification and polymerization. However, noticeable differences between them can be distinguished in the images. As illustrated in Figure 4D, the imprinted polymer layers with uniform thickness were densely combined with the surface of mesoporous carriers. As expected, we can posit that the novel MT-MIPs based on mesoporous carriers were successfully prepared. Moreover, the thin imprinted layers could be beneficial for the mass transfer between templates and the surface of SBA-15@MT-MIPs.

**Figure 5:**
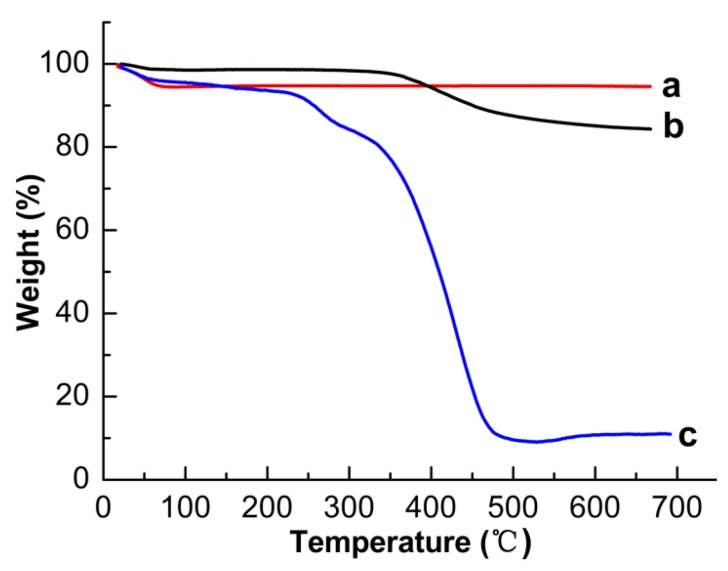
Thermogravimetric weight loss curves of (a) SBA-15; (b) SBA-15-MPS; (c) SBA-15@MT-MIPs.

To investigate the thermostability and solid content of the materials, Figure 5 shows the TGA curves of SBA-15, SBA-15-MPS and SBA-15@MT-MIPs in the temperature range from 25 °C to 700 °C. As shown in Figure 5(a), The TGA curve of SBA-15 had only one weight loss stage in the temperature below 100 °C, which was assigned to the release of physically and chemically adsorbed water in SBA-15. Figure 5(b) illustrates that SBA-15-MPS had two weight loss steps in the range of 25–700 °C. The first step below 100 °C was the release of physically adsorbed water and solvent residues in SBA-15. The second weight loss step showed around 14% weight loss in the temperature range of 100–550 °C, which corresponded to the degradation of the MPS silane coupling agent. Analogously, in the TGA curve of SBA-15@MT-MIPs (Figure 5(c)), the first weight loss step corresponded to the surface adsorbed water. The next much higher weight loss (around 82%) was observed from 100 °C to 482 °C, which could be assigned to the removal of the organic content in the imprinted layers. Meanwhile, it was confirmed that imprinted polymer layers were successfully synthesized.

N_2_ adsorption-desorption analysis was used to evaluate the porosity changes of the mesoporous SBA-15 by the introduction of MPS and MIP layers. The surface areas were measured with Brunauer-Emmett-Teller (BET) theory. Total pore volumes and pore size distributions were obtained based on the adsorption branch of the isotherm with the Barrett-Joyner-Halenda (BJH) method. The results were shown in Table 2. After the attachment of MPS, the BET surface area, pore volume and pore size of SBA-15-MPS decreased slightly compared with SBA-15. However, the surface area (*S*_BET_), pore volume (*V*_T_) and pore size (*D*_P_) of SBA-15@MT-MIPs decreased drastically to 140.2 m^2^·g^−1^, 0.1018 cm^3^·g^−1^ and 34.28 Å, respectively. These changes provided proof that the imprinted layers existed in the nanopores of SBA-15, which has been reported elsewhere [48].

### 3.3. Investigation on the Performance of SBA-15@MT-MIPs

#### 3.3.1. Study of Binding Properties

The binding properties of polymers towards Rb_1_, Rg_1_ and R_1_ was investigated by the static adsorption equilibrium experiment and adsorption kinetic studies.

**Figure 6:**
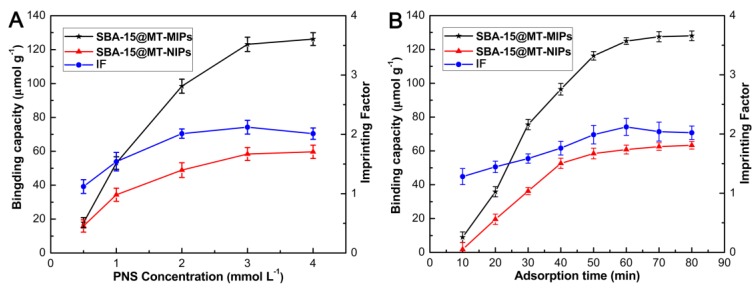
(**A**) Adsorption isotherms and imprinting factors for SBA-15@MT-MIPs and SBA-15@MT-NIPs with different concentrations of the mixture solution of Rb_1_, Rg_1_ and R_1_, where *IF* was defined as *IF* = *Q_MIP_*/*Q_NIP_*; (**B**) Adsorption kinetic behaviors of SBA-15@MT-MIPs and SBA-15@MT-NIPs for Rg_1_, Rb_1_, R_1_ with corresponding imprinting factors.

Binding isotherms are a measure of the concentration dependent recognition behavior of a system. As shown in Figure 6A, the adsorption capacities of SBA-15@MT-MIPs and SBA-15@MT-NIPs for Rb_1_, Rg_1_ and R_1_ increased rapidly in the beginning stages and later slowed down with the increasing of initial concentration. Then the adsorption tended to be stable gradually and become constant at 3 mmol·L^−1^, due to the saturation of recognition sites. The saturated adsorption capacities of SBA-15@MT-MIPs and SBA-15@MT-NIPs for Rb_1_, Rg_1_ and R_1_ were calculated to 123.11, 58.41 μmol·g^−1^, respectively. The *IF* of the SBA-15@MT-MIPs for templates (Rb_1_, Rg_1_ and R_1_) was also found satisfactory (*IF* = 2.11). Evidently, the amount of Rb_1_, Rg_1_ and R_1_ bonded on the SBA-15@MT-MIPs cavities was larger than that on the corresponding SBA-15@MT-NIPs at all the concentrations studies. This obvious preferential adsorption was ascribed to the SBA-15@MT-MIPs containing special binding sites complementary in size and shape to the template molecules and the existence of hydrogen bonds between the functional groups and template molecules. 

The adsorption kinetic study can provide valuable information about both the binding and the rate-controlling mechanism. As indicated in the Figure 6B, the adsorption rate of the SBA-15@MT-MIPs towards the mixture of Rb_1_, Rg_1_ and R_1_ increased sharply in the first 30 min and then slowed down gradually to reach equilibrium within 60 min. The maximum absorption capacity of SBA-15@MT-MIPs for Rb_1_, Rg_1_ and R_1_ was 123.11 μmol·g^−1^ and the value of *IF* was 2.12. In addition, the dynamic curve of SBA-15@MT-NIPs showed a similar trend with that of SBA-15@MT-MIPs but the lower adsorption amount. These could occur because the specific recognition sites of SBA-15@MT-MIPs were generated in the molecular imprinting process and most of the binding sites were located in the proximity of the interior surface. So, target species could easily diffuse into the recognition sites of SBA-15@MT-MIPs, leading to the higher binding capacity and faster mass transfer rate. 

#### 3.3.2. Molecular Selectivity of the SBA-15@MT-MIPs

**Figure 7:**
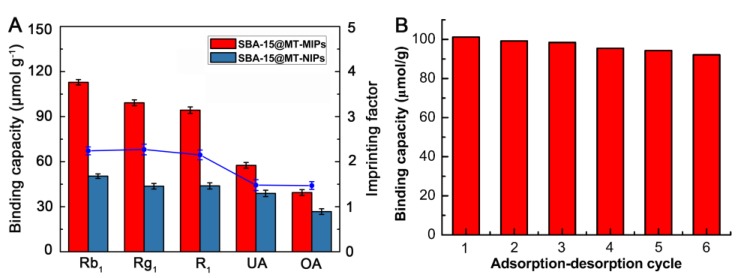
(**A**) Selective adsorptions of SBA-15@MT-MIPs and SBA-15@MT-NIPs towards Rb_1_, Rg_1_, R_1_, UA and OA standard solution with corresponding imprinting factors; (**B**) Reusability of SBA-15@MT-MIPs.

To measure the specificity of SBA-15@MT-MIPs, UA and OA were chosen as the reference compounds. The molecular structures of Rb_1_, Rg_1_, R_1_, UA and OA can be seen in Figure 2. From Figure 7A, we can see that the *Q* and *IF* of the SBA-15@MT-MIPs towards the templates of Rb_1_, Rg_1_ and R_1_ were significantly higher than those of the structural analogs, UA and OA. However, the binding capacities of SBA-15@MT-NIPs for the five analytes were almost the same and all lower than those of SBA-15@MT-MIPs. These results confirmed that SBA-15@MT-MIPs possessed higher selectivity and affinity toward Rb_1_, Rg_1_ and R_1_ owing to the existence of the specific recognition sites between the monomers and template molecules. As the competitive structural analog UA and OA, although UA and OA are similar to Rb_1_, Rg_1_ and R_1_ in the molecular volume and structures, the recognition sites were not complementary to the structural analogs. In other words, the memory of specific functional groups also played an important part in conformation memory [49].

#### 3.3.3. Reusability of SBA-15@MT-MIPs

The stability of SBA-15@MT-MIPs was evaluated by comparing the adsorption capacity towards Rb_1_, Rg_1_ and R_1_ at each cycle. As shown in Figure 7B, the adsorption capacity decreased slowly with an increase in cycle times. After six adsorption-desorption cycles, the adsorption capacity was about 9.09% less. The slight decrease in adsorption capacity mainly resulted from the partial destruction of the recognition sites during the elution procedure. The results indicated that recognition, interaction and adsorption processes occurred reversibly and the polymers had excellent stability and acceptable reusability. So, it could be used for practical applications.

### 3.4. Separation and Determination of Rb_1_, Rg_1_ and R_1_ in Real Samples

#### 3.4.1. Optimization of the MISPE Protocols

**Figure 8:**
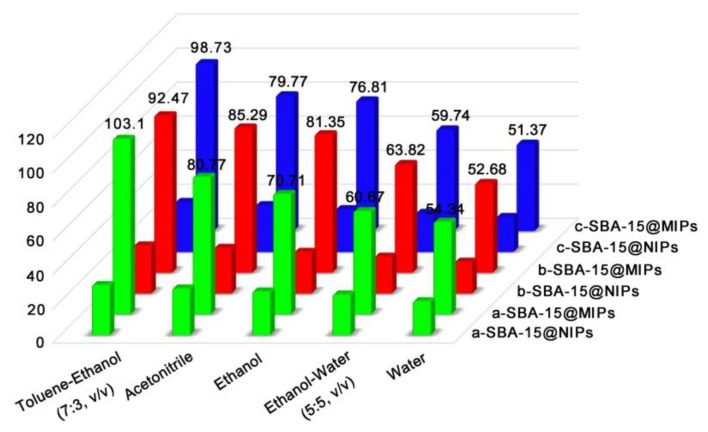
Recoveries of R_1_, Rg_1_, Rb_1_ in the washing fractions of SBA-15@MT-MIPs and SBA-15@MT-NIPs with different washing solvents (a: R_1_, b: Rg_1_ and c: Rb_1_).

The washing procedure is a necessary procedure which could minimize the interferences for the analysis step and activate the binding sites of SBA-15@MT-MIPs for maximizing their interaction with the target analytes. Generally, low-polar solvent was preferred as washing solvent such as chloroform and toluene to disrupt the nonspecific binding between the polymers and matrix components [50]. Initially SPE cartridges containing 500 mg of SBA-15@MT-MIPs and SBA-15@MT-NIPs were loaded with 5 mL of 3.0 mmol·L^−1^ the mixture solution of Rb_1_, Rg_1_ and R_1_ in blank rat plasmas and then 5 mL of each washing solvent was applied. For optimizing the condition of the washing step, the various washing solvents, such as toluene-ethanol (7:3, *v*/*v*), acetonitrile, ethanol, ethanol-water (5:5, *v*/*v*) and water were studied. The results were shown in Figure 8. It can be observed that at least 70% of the Rb_1_, Rg_1_ and R_1_ were washed off from the SBA-15@MT-NIPs cartridge after it was washed by above five washing solvents, because there was only physical adsorption for SBA-15@MT-NIPs. However, when using toluene-ethanol (7:3, *v*/*v*) as washing solvent, the impurities in samples could be mostly cleaned up and the recoveries of Rb_1_, Rg_1_ and R_1_ were more than 92.47% in MISPE. Following are the reasons. Firstly, toluene-ethanol (7:3, *v*/*v*) can induce the formation of hydrogen bonding between templates (Rb_1_, Rg_1_ and R_1_) and functional monomers and at the same time interfering compounds were easier washed. Secondly, the specific interaction between the templates and SBA-15@MT-MIPs was easily disrupted by highly polar solvents (acetonitrile, ethanol, ethanol-water (5:5, *v*/*v*) and water). Thus, toluene-ethanol (7:3, *v*/*v*) as the optimal washing solution was chosen for running the subsequent experiments.

The selection of eluent plays a key role in excellent sensitivity and precision. It was reported that methanol-acetic acid (9:1, *v*/*v*) exhibits the good recovery [51,52]. The reason may be that acetic acid can break the formation of hydrogen bonding between template and functional monomer and template desorption was easier [50]. Thus, we applied 5 mL of methanol-acetic acid (9:1, *v*/*v*) as eluent agent.

#### 3.4.2. Validation Assay

Under optimized conditions of MISPE coupled with HPLC, the linearity, the detection range (LODs) and limit (LOQs) were investigated. The results were shown in Table 3. Calibration curves were established for R_1_, Rg_1_ and Rb_1_ in rat spiked plasmas over a concentration range of 2.3–47.3 ng·mL^−1^ with benign regression coefficients R^2^ > 0.995. The LODs and LOQs were determined based on the signal to noise (S/N) ratio of 3 and 10, respectively. The LODs and LOQs of the proposed method for R_1_, Rg_1_ and Rb_1_ were in the range of 0.63–0.75 ng·mL^−1^ and 2.1–2.5 ng·mL^−1^, respectively. The relative standard deviations (RSDs) for R_1_, Rg_1_ and Rb_1_ were 2.5%, 3.0% and 2.2%, respectively, which was repeated measurement for five times with 10 ng·mL^−1^ of R^1^ and 20 ng·mL^−1^ of Rg_1_ and Rb_1_, respectively. It was demonstrated the developed method had a satisfactory reproducibility.

The accuracy and practicability of the method were verified by spiked recovery and summarized in Table 4. Since no Rb_1_, Rg_1_ and R_1_ in the collected the rat plasma samples of was detectable by the proposed method, spiked recoveries were carried out of the samples spiked with 2.5–16 ng·mL^−1^ R_1_, Rg_1_ and Rb_1_ to evaluate the developed method. As can be seen, the average recoveries at the middle concentration of the developed method for R_1_, Rg_1_ and Rb_1_ were 101.4%, 93.4% and 94.3%, respectively, with RSD < 4.2%. The results indicated a suitable improvement in the recovery and accuracy even at the low concentration was found and suggested that SBA-15@MT-MIPs can directly be used for selective adsorption and detection of R_1_, Rg_1_ and Rb_1_ in biological samples. In addition, we had compared the prepared MIPs as SPE sorbents with commercial C_18_ column and polydimethylsiloxane stir bar (PDMS-SB) to validate the absorptive recovery of the prepared MIPs in our initial studies [53,54], which showed that the recoveries of the targets on MIPs were higher than that of commercial column. Therefore, SBA-15@MT-MIPs can be directly used for selective separation and determination of trace saponins in biological samples.

#### 3.4.3. Application to the Determination of Trace Rb_1_, Rg_1_ and R_1_ in Real Samples 

**Figure 9:**
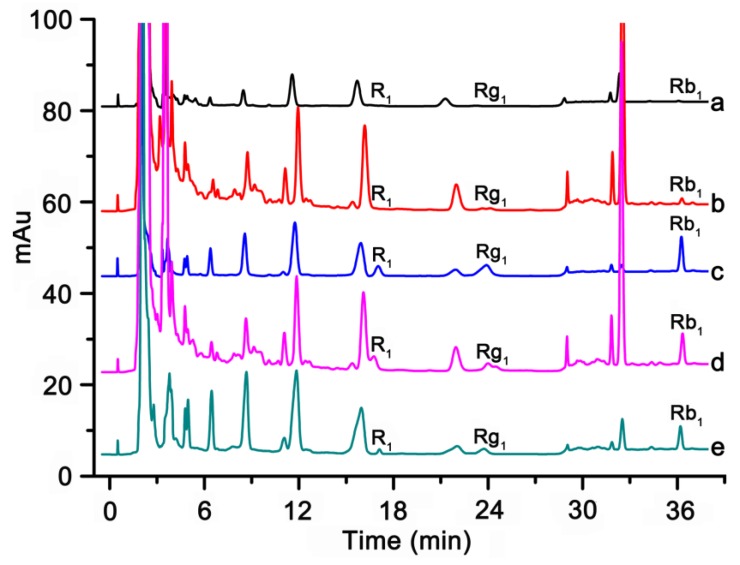
Chromatograms of Rb_1_, Rg_1_, R_1_ in rat plasma samples (a) by direct injection without enrichment; (b) the washing solution protocol obtained from MISPE; (c) the elution solution protocol obtained from MISPE; (d) the washing solution protocol obtained from NISPE; (e) the elution solution protocol obtained from NISPE.

To evaluate the enrichment and impurity-removing property of the MISPE column and whether the method is suitable for low concentration detection, the proposed method was applied to the analysis of Rb_1_, Rg_1_ and R_1_ in rat plasma samples and the results were shown in Figure 9. As shown in Figure 9(a), because of extremely low concentration, the analytes were hardly detectable without performing the separation and enrichment of process. Most of the matrix components only with few analytes were eliminated efficiently after being separated by MISPE column (Figure 9(b)). As shown in Figure 9(c), R_1_, Rb_1_ and Rg_1_ were remarkably concentrated and the interfering peaks arising from complex biological matrices were suppressed, indicating the remarkable preconcentration ability and desired specific recognition of the novel MISPE sorbents to R_1_, Rb_1_ and Rg_1_. However, Figure 9(d) showed that most of the analytes and matrix components were washed out from the non-imprinted polymer solid-phase extraction (NISPE) column and few analytes were detected in the elution solution obtained from NISPE (Figure 9(e)). In addition, the amounts of R_1_, Rb_1_ and Rg_1_ that were detected from the rat plasma samples were 2.5 ng·mL^−1^, 4.0 ng·mL^−1^ and 4.6 ng·mL^−1^, respectively. Finally, the target analytes in the elution solution obtained from MISPE sorbents have been structurally identified based on HPLC-MS and the experimental results were listed in Table 5.

As could be observed from the chromatograms, the sensitivities of Rb_1_, Rg_1_ and R_1_ in plasma samples were greatly enhanced with the MISPE coupled with HPLC-UV analysis. This process did not require special instrumentation, consumes much less toxic organic solvent and has a good clean-up and concentration effect for the analytes. These results demonstrated that the MISPE coupled with HPLC offers a suitable method for selective separation and determination of trace saponins in biological samples.

## 4. Conclusions

In the present study, the innovative multi-template imprinted polymers based on SBA-15 have been prepared by combining the merits of surface imprinting technique and mesoporous material. The obtained SBA-15@MT-MIPs exhibited good characteristics such as excellent adsorption capacity, fast equilibrium kinetics and favorable selectivity towards Rb_1_, Rg_1_ and R_1_. Moreover, low LOD and good recovery were obtained, indicating the great applicability for selective separation of specific analytes from complex samples. Finally, the reliable and simple approach has been successfully employed for the efficient remove and quantitative enrichment of the trace level of PNS from plasma samples simultaneously in a single run. In conclusion, the developed method will find wider applications in diverse clinical and toxicological laboratories for the routine analysis of PNS in biological samples.

## Figures and Tables

**Table 1:** The preparation of SBA-15@MT-MIPs under different conditions.

Polymer	Functional Monomer	Molar Ratio ^a^	Solvents	*Q* (µmol·g^−1^)	*IF* ^b^
SBA-15@MT-MIPs-1	4-VP	1:4:16	ethanol	75.51	1.74
SBA-15@MT-MIPs-2	MAA	1:4:16	ethanol	81.30	1.46
SBA-15@MT-MIPs-3	AM	1:4:16	ethanol	108.9	2.11
SBA-15@MT-MIPs-4	AM	1:3:12	ethanol	68.71	1.02
SBA-15@MT-MIPs-5	AM	1:5:20	ethanol	93.00	2.03
SBA-15@MT-MIPs-6	AM	1:6:24	ethanol	94.50	2.07
SBA-15@MT-MIPs-7	AM	1:4:16	tetrahydrofuran	31.57	1.82
SBA-15@MT-MIPs-8	AM	1:4:16	ethanol-acetonitrile (2:3)	93.22	1.04

^a^ The molar ratio refers to template:functional monomer:cross-linker; ^b^ Imprinting factor (*IF*) = *Q_MIP_*/*Q_NIP_*.

**Table 2:** Porosities of polymers determined by N_2_ adsorption-desorption analysis.

Sample	*S*_BET_ (m^2^·g^−1^)	*V*_T_ (cm^3^·g^−1^)	*D*_P_ (Å)
SBA-15	631.7	1.128	68.79
SBA-15-MPS	436.9	0.7433	64.52
SBA-15@MT-MIPs	140.2	0.1018	34.28

**Table 3:** The performance parameters of the proposed method (*n* = 5).

Compounds	Linear Range (ng·mL^−1^)	R^2^	LOD (ng·mL^−1^)	LOQ (ng·mL^−1^)	RSD (%)
R_1_	2.3–19.8	0.996	0.63	2.1	2.5
Rg_1_	3.7–47.3	0.995	0.75	2.5	3.0
Rb_1_	3.2–44.5	0.995	0.69	2.3	2.2

**Table 4:** Spiked recoveries of R_1_, Rg_1_ and Rb_1_ in plasma samples (*n* = 3).

Compounds	Concentration Taken (ng·mL^−1^)	Found (ng·mL^−1^)	Recovery (%)	RSD (%)
R_1_	2.5	2.6	104.3	4.2
	5.0	5.1	101.4	4.0
	10.0	9.9	99.0	3.8
Rg_1_	4.0	3.8	95.0	3.7
	8.0	7.5	93.4	3.6
	16.0	15.2	95.0	3.3
Rb_1_	3.5	3.3	94.3	3.6
	7.0	6.6	94.3	3.3
	14.0	13.5	96.4	3.5

**Table 5:** The detected chromatographic and spectrometric data of the separated compound in the HPLC chromatograms.

Peak	Compounds	Formula	Retention Time (min)	[M + H]^+^ (*m*/*z*)	Reference
1	R_1_	C_47_H_80_O_18_	16.88	932.5	[55]
2	Rg_1_	C_42_H_72_O_14_	24.34	824.5	[56]
3	Rb_1_	C_54_H_92_O_23_	36.34	1109.31	[57]
